# High‐Efficacy and Polymeric Solid‐Electrolyte Interphase for Closely Packed Li Electrodeposition

**DOI:** 10.1002/advs.202003240

**Published:** 2021-01-29

**Authors:** Siyuan Li, Qilei Liu, Weidong Zhang, Lei Fan, Xinyang Wang, Xiao Wang, Zeyu Shen, Xiaoxian Zang, Yu Zhao, Fuyuan Ma, Yingying Lu

**Affiliations:** ^1^ State Key Laboratory of Chemical Engineering Institute of Pharmaceutical Engineering College of Chemical and Biological Engineering Zhejiang University Hangzhou 310027 China; ^2^ Institute of Chemical Process Systems Engineering School of Chemical Engineering Dalian University of Technology Dalian 116024 China; ^3^ Key Laboratory of Solar Energy Utilization & Energy Saving Technology of Zhejiang Province Zhejiang Energy R&D Institute Co., Ltd. Hangzhou 311121 China

**Keywords:** anode‐free configuration, high‐efficacy, LiDFOB salt, practical Li‐metal batteries, solid‐electrolyte interface

## Abstract

The industrial application of lithium metal anode requires less side reaction between active lithium and electrolyte, which demands the sustainability of the electrolyte‐induced solid‐electrolyte interface. Here, through a new diluted lithium difluoro(oxalato)borate‐based (LiDFOB) high concentration electrolyte system, it is found that the oxidation behavior of aggregated LiDFOB salt has a great impact on solid‐electrolyte interphase (SEI) formation and Li reversibility. Under the operation window of Cu/LiNi_0.8_Co_0.1_Mn_0.1_O_2_ full cells (rather than Li/Cu configuration), a polyether/coordinated borate containing solid‐electrolyte interphase with inner Li_2_O crystalline can be observed with the increasing concentration of salt, which can be ascribed to the reaction between aggregated electron‐deficient borate species and electron‐rich alkoxide SEI components. The high Li reversibility (99.34%) and near‐theoretical lithium deposition enable the stable cycling of LiNi_0.8_Co_0.1_Mn_0.1_O_2_/Li cells (*N*/*P* < 2, 350 Wh kg^−1^) under high cutoff voltage condition of 4.6 V and lean electrolyte condition (*E*/*C* ≈ 3.2 g Ah^−1^).

## Graphical abstract

High‐efficacy and polymeric solid‐electrolyte interphase is in situ formed on lithium metal anode by using a new diluted lithium difluoro(oxalato)borate‐based (LiDFOB) high‐concentration electrolyte. The outer SEI layer is an amorphous polyether/tri‐coordinated borate polymeric organic phase, while the inner layer contains robust Li_2_O inorganic crystalline. As‐fabricated cells deliver a high Li reversibility of 99.34% and long full‐cell lifetime under ≈350 Wh kg^−1^.

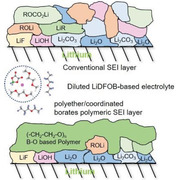

## 1 Introduction

The emerging electric vehicles and large grids require advanced high‐energy‐density rechargeable batteries to utilize aggressive cathode and anode.^[^ ^1^, ^2^, ^3^, ^4^ ^]^ Lithium metal is considered to be one of the promising anode materials due to its high theoretical specific capacity (3860 mA h g^−1^) and the most negative electrochemical potential (−3.04 V vs standard hydrogen electrode).^[^ ^5^, ^6^, ^7^ ^]^ Replacing graphite with lithium metal anode to pair with high voltage/high capacity cathode e.g. Ni‐rich LiNi_0.8_Co_0.1_Mn_0.1_O_2_ (NCM811) and high‐voltage LiCoO_2_ (LCO) can improve cell energy density to 400 Wh kg^−1^.^[^ ^8^, ^9^ ^]^

However, it is still a great challenge to commercialize lithium metal batteries (LMBs) because of its unstable cycling. The intrinsic lowest redox potential of Li brings a higher reactivity, leading to uncontrolled interfacial reactions and unstable solid‐electrolyte interphase (SEI).^[^ ^10^ ^]^ The poor SEI layer induces further whisker‐like, high‐tortuosity Li deposition, where internal Li is more likely to lose electronic connection while stripping, rendering a low coulombic efficiency (CE).^[^ ^11^ ^]^ In order to achieve the same lifetime compared to graphite‐based cells, a high CE of >99.9% is required on Li metal side in LMBs to guarantee the adequate lithium source during cycling.^[^ ^12^ ^]^ Although lots of modifications have been made in the early stage, such as 3D skeleton, artificial interface layer, electrolyte additives and solid‐state electrolyte, etc., none of them can maintain a high CE due to the continuous reaction between active Li and electrolyte.^[^ ^13^, ^14^, ^15^, ^16^, ^17^ ^]^ Especially under the practical condition including limited negative to positive electrode capacity ratio (*N*/*P* ratio) and small electrolyte weight to cathode capacity ratio (*E*/*C* ratio), the large volume change and additive exhaustion lessen the lifespan of practical Li metal batteries to 30 cycles. Therefore, it is significant to minimize the irreversible lithium loss in every cycle by the rational design of the electrode/electrolyte interface, for which the electrolyte‐induced SEI layer needs to be carefully modified.

An ideal SEI film must be able to be reused during the cycling to reduce the formation of new SEI and the consumption of necessary resources (active lithium, electrolyte, etc.). Improving the uniformity of SEI (e.g., Li_2_O‐amorphous double‐layer structure SEI) can facilitate even local current buildup. In addition, boosting mechanical strength and ductility enables the electrode to endure the large volume expansion during cycling.^[^ ^18^, ^19^ ^]^ Rational designing of in situ formed SEI always involved in modifying electrolyte components or additives, in which some functional additives like LiNO_3_, Cs^+^, SnTFSI are considered to be a good way to improve CE temporarily, whereas the additive exhaustion needs to be concerned in lean condition.^[^ ^20^, ^21^, ^22^, ^23^, ^24^, ^25^ ^]^ Therefore, adjusting the solvent components and concentration of lithium salts to improve the stability of SEI becomes a new approach toward high‐voltage/long‐lifespan LMBs. For example, constructing an all‐fluorinated electrolyte, LiDFOB‐LiBF_4_ dual‐salt electrolyte or highly concentrated electrolyte.^[^ ^26^, ^27^, ^28^, ^29^ ^]^ Recently, the LiFSI containing concentrated electrolyte is demonstrated to obtain a high CE of >98.5% at Li/Cu configuration. The concentrated lithium salt can reduce the effect of convective mass transfer as well as the number of free solvent molecules, forming a dense, F‐dominated decomposition. Additionally, finding new ways to modify the in situ SEI, like developing a polymer‐like organic phase, is also critical for basic research and industrialization, which has been rarely reported.

**Figure 1:**
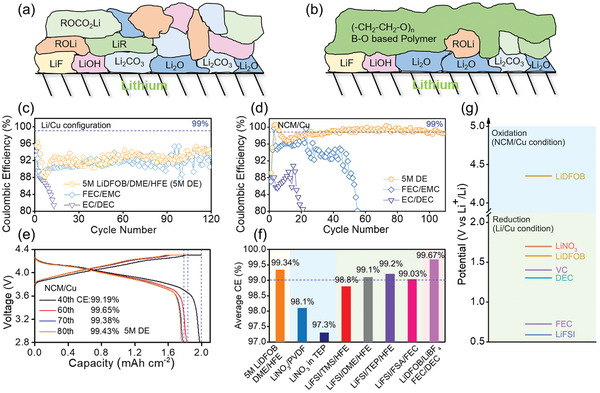
Property of high‐efficacy SEI induced by 5 m LiDFOB/DME/HFE (5 m DE) electrolyte under NCM/Cu configuration and its Li reversibility. a,b) Schemes of conventional electrolyte‐reduction monomer SEI layer and high‐efficacy polyether‐like SEI. c,d) Li reversibility in Li/Cu and NCM/Cu configuration of three typical electrolytes (note: CE in NCM/Cu will be a more reliable indicator for true Li reversibility in full cell operation). e) Voltage curves of NCM/Cu cell using 5 m DE electrolyte during 40th–80th cycles. f) Summary of Li reversibility of state‐of‐the‐art electrolyte system. g) Experimental reduction and oxidation potential of various electrolyte components reported in the literature.

Conventional electrolyte‐reduction SEI generally consists of two layers, including the inner inorganic layer close to the Li surface (such as Li_2_O, LiF, LiOH, and Li_2_CO_3_) and outer organic layer with higher oxidation states (such as ROCO_2_Li, ROLi, and LiR), which always show a mosaic‐like structure and low sustainability during long cycling (**Figure** **1**a).^[^ ^30^ ^]^ Herein, we report an high‐efficacy, polymer‐like SEI for highly stable Li metal anodes by utilizing a new diluted high concentration lithium difluoro(oxalato)borate/1,2‐dimethoxyethane/1,1,2,2‐tetrafluoroethyl‐2,2,3,3‐tetrafluoropropylether (LiDFOB/DME/HFE) electrolyte under full cell configuration (2.8–4.4 V). LiDFOB salt undergoes different decomposition mechanisms under conditions of Li/Cu and LiNi_0.8_Co_0.1_Mn_0.1_O_2_/Cu (denoted as NCM/Cu) due to the different operating voltage windows. Through DFT calculation, concentrated LiDFOB salts tend to be oxidized to form electron‐deficient B products under high voltage condition, and further react with the electron‐rich components (RO‐Li, alkyl carbonates) in conventional SEI to form polyether/tri‐coordinated borates polymeric organic phase (Figure 1b). Cryoelectron microscopy observation reveals that the SEI consists of two layers, in which the outer layer is an amorphous polymeric organic phase and the inner side is Li_2_O crystallization. This sustainable and robust SEI can be reused during lithium plating/stripping, exhibiting an ultrahigh coulombic efficiency of 99.34% during 40–110 cycles in NCM/Cu configuration. The high Li reversibility enables excellent cycling of Li/LiNi_0.8_Mn_0.1_Co_0.1_O_2_ for 129 cycles with 80% capacity retention under highly challenging conditions (≈350 Wh kg^−1^ at cell‐level, cathode loading >4.5 mAh cm^−2^, negative to positive electrode capacity ratio of 2.0 and electrolyte weight to cathode capacity ratio of 3.2 g Ah^−1^).

## 2 The Li Reversibility of Diluted LiDFOB‐Based Electrolyte in NCM/Cu Configuration

Columbic efficiency is an important indicator for long lifetime cycling. In this part, we found that 1) CE in NCM/Cu will be a more reliable indicator for true Li reversibility in full cell operation, especially for LiDFOB‐based electrolyte, whose oxidation behavior can regulate the SEI layer on Li metal. 2) Revealed by NCM/Cu cell, high concentration of LiDFOB is a good method to improve the Li reversibility in ester, ether and phosphate‐based electrolyte. Considering the maximum solubility of LiDFOB in DME is 7 m (Figure S1, Supporting Information), three different concentrations of 1 , 3 , and 5 m LiDFOB/DME solutions were prepared, among which 3 and 5 m solutions were diluted by HFE cosolvent to 1 m (denoted as 5 and 3 m diluted electrolyte, DE) to obtain a better separator wettability. The corresponding viscosity data of different electrolytes are listed in Table S1 (Supporting Information). Five kinds of electrolytes containing 1 m LiPF_6_ in EC/DEC (v:v = 1:1), 1 m LiPF_6_ in FEC/EMC (v:v = 3:7), 1 m LiDFOB in DME, 3 m DE and 5 m DE were first examined to evaluate the disparity of their Li reversibility between Li/Cu and NCM/Cu cell. Considering the anode‐free property of NCM/Cu, the Coulombic inefficiency (CI, CI = 1‐CE) in full cell can be attributed to the irreversible Li loss in forming SEI, CEI and dead lithium. Therefore, it is reasonable to regard the CE in NCM/Cu as lithium reversibility. Two conventional LiPF_6_‐based electrolytes show similar CE in both Li/Cu and NCM/Cu cells. For EC/DEC, CE in cells show a rapid drop below ≈80% within 20 cycles owing to the low Li utilization in SEI (Figure 1c,d). Adding FEC into carbonate electrolyte will improve the Li utilization. However, the average CE in both Li/Cu and NCM/Cu is still inadequate for practical application (≈92–94%). Interestingly, the situation becomes different for LiDFOB‐based electrolytes. LiDFOB/DME electrolyte without dilution exhibits the ultralow CE in Li/Cu cell due to the poor stability of solvent against Cu foils. Adding the co‐solvent HFE can promote the electrolyte stability, which is reflected by improving CE to 92% in 3 m DE and 5 m DE (Figure S2, Supporting Information). However, this kind of CE does not meet the requirements for commercialization (e.g., CE > 99.9% for graphite). The corresponding voltage curves are presented in Figure S3 (Supporting Information). When NCM/Cu configuration is used, the 5 m DE electrolyte displays an increasing CE to 99% in 40 cycles and maintains an average Li reversibility of ≈99.34% in the following 70 cycles (Figure 1d). The tremendous improvement can be attributed to the oxidation behavior of concentrated LiDFOB, which will be introduced later. Different concentration of LiDFOB/DME/HFE electrolytes are also detected in NCM/Cu cell and an improved CE of ≈94% and ≈96% can be observed (Figure S4, Supporting Information). The detailed voltage curves of different electrolytes in NCM/Cu are displayed in Figure 1e and Figure S5 (Supporting Information). With 5 m DE electrolyte, the cell shows high Li reversibilities of 99.19%, 99.65%, 99.38%, and 99.43% in 40th, 60th, 70th, and 80th cycles, respectively. The normalized cycling performance of anode‐free NCM/Cu cell using 5 m DE is shown in Figure S6 (Supporting Information), which is one of the best reported cycling stabilities with anode‐free configuration, high areal capacity and high current density. Apart from ether, this high concentration LiDFOB‐based method can be further used in ester and phosphate‐based electrolyte, as displayed in Note S1 in the Supporting Information. This result also proves that, compared with conventional LiPF_6_‐based electrolyte, the CE in Li/Cu cell is not completely equal to the utilization rate of lithium in the actual full battery cycling, which demonstrates that the aggregate LiDFOB can regulate the Li deposition under the voltage window of NCM/Cu. The detailed CEs of different electrolyte system are listed in Figure 1f. Although the prevailing view is that using LiNO_3_ additive is beneficial to CE improvement, the average CE of this method remains only ≈98.5%, which cannot meet the requirement of commercialization.^[^ ^22^, ^31^ ^]^ LiFSI‐based high concentration electrolyte further breaks through the target of 99% while LiDFOB/LiBF_4_ dual‐salt electrolyte show an ultrahigh CE above 99.6% for 200 cycles.^[^ ^32^, ^33^, ^34^, ^35^, ^36^ ^]^ Before being applied in full cell, the major difference between Li/Cu and NCM/Cu cells should be explained. The current Li/Cu measurement is conducted based on the voltage window of 0–1 V, in which most of electrolyte components are reduced before LiDFOB salt, forming a solvent/salt‐reduced SEI. However, the voltage window is widened to 2.8–4.4 V in NCM/Cu condition, where the oxidation effect of lithium salts on anode should be taken into account (Figure 1g).

## 3 Polyether‐Like Solid‐Electrolyte Interphase and Solvation Structure

**Figure 2:**
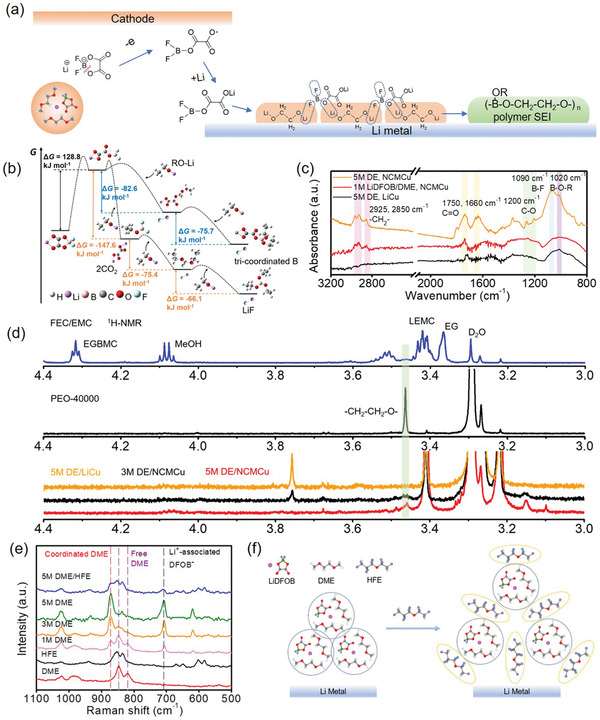
Possible reaction mechanism and solvation structure. a) Proposed oxidative decomposition and condensation polymerization of LiDFOB with alkoxides in NCM/Cu cell at electrochemical window of 2.8–4.4 V. b) Thermodynamic trend of the reaction of LiDFOB oxidation products with electron‐rich alkoxides through DFT calculation. c) ATR‐FTIR spectra of SEI residues induced by different electrolytes on Cu foils. d) NMR spectra collected from SEIs generated in electrolyte solutions of 1 m LiPF_6_ in FEC/EMC, 5 m DE/NCMCu, 5 m DE/LiCu, 3 m DE/NCMCu after 30 cycles and PEO‐40000: ^1^H NMR, DMSO‐d_6_. e) Raman spectra of different solvents and electrolytes. f) The scheme of the solvation structure in the 5 m LiDFOB/DME/HFE.

The different behaviors in Li/Cu and NCM/Cu configuration imply that LiDFOB salt may exhibits different decomposition mechanisms in oxidation and reduction processes, which further influence the property of SEI layer. Under the low voltage window of 0–1 V, LiDFOB prefer to get the electron from Li metal and exhibits a B—O or B—F bond‐cleavage behavior, forming an low‐energy electron‐rich species and LiF (Figure S7, Supporting Information), which is consistent with the observation of Lucht and co‐workers that nanostructured LiF particles is generated resulting from the presence of oxalate based capping agents.^[^ ^37^, ^38^, ^39^ ^]^ However, when electrochemical window broadens to 2.8–4.4 V, the oxidation of LiDFOB and further reaction become the main step of SEI formation. The compound can easily lose an electron on the NCM cathode surface at high potentials (oxidation potential of LiDFOB is ≈4.3 V vs Li/Li^+^). The LiDFOB rings opening, and forms an electron‐deficient difluoroborane species (or radical), which subsequently react with the electron‐rich alkoxides (e.g., LiOCH_2_CH_2_OLi, a probable SEI film component in ether) or carbonates in SEI to form a –(BOR–O–CH_2_–CH_2_–O)*_n_–* like polymeric component (**Figure** **2**a).^[^ ^40^, ^41^, ^42^ ^]^ In this work, we just calculate the ligand exchange reactions through different B–F or B–O cleavage pathway using lithium ethoxide as routine SEI substance from a thermodynamic point of view. As shown in Figure 2b, energy absorption is essential for the first B–O ring opening, which means the external energy like high voltage electric field may be necessary for further reaction. After that, difluoroborane species can easily react with lithium ethoxide, reflecting as the decrease of Gibbs free energy in both B–F and B–O cleavage pathway. Moreover, the B—O bond cleavage further forms 2CO_2_, which is more preferable than B–F pathway due to the larger Gibbs free energy drop.^[^ ^38^ ^]^ It is assumed that the as‐formed oligomeric and polymer species in the SEI makes the surface film elastic, which is beneficial for uniform Li deposition and dendrite suppression.

The detailed decomposition products in different configurations were further detected by ATR‐FTIR measurement (Figure 2c; Figure S8, Supporting Information). Before the test, the electrodes were carefully washed with DME solution and dried in vacuum for several hours to ensure that the detected peaks fully come from the components in SEI layer rather than remaining residue. According to the DFT calculation, LiDFOB salt prefers to lose F atom to form LiF under reduction condition while it will react with electron‐rich components like ROLi to form organic products under oxidation condition, which shows different characteristic peaks in FTIR spectra. In 5 m DE/LiCu configuration, the peaks located in 1750 and 1660 cm^−1^ can be ascribed to the C=O stretching vibrations. Meanwhile, a board peak from 900 to 1000 cm^−1^ can be attributed to the B‐containing decomposition product of LiDFOB in the SEI layer. The similar peaks are also observed in 1 m LiDFOB/DME NCM/Cu configuration except the obvious dual‐peak in 2925–2850 cm^−1^, which corresponds to the –CH_2_– stretching vibration from some accumulated dimer or oligomer SEI residue. The characteristic peaks in 5 m DE/NCMCu configuration become more obvious due to the decomposition of LiDFOB in high‐voltage condition. As can be seen in Figure 2c, B–O–R and B–F linkages (1020 and 1090 cm^−1^) related to tri‐coordinated borates in SEI become more intensive, reflecting that electron‐deficient B species will react with other SEI components like lithium semicarbonates or lithium alkoxide to form stable polymer. The direct evidence is that the peaks in ≈1200 cm^−1^ of C–O in aliphatic ethers can be clearly observed, which can only be detected in high‐concentration LiDFOB electrolyte.^[^ ^43^ ^]^ Considering that the organic species with small molecular weight has been completely removed, the remaining C–O, –CH_2_–, and tri‐coordinated B species can be ascribed to the highly polymerized B crosslink polyether in SEI, which is in accordance with the presence of –(CH_2_–CH_2_–O)*_n_*– and B‐O species in XPS test. It is likely that with the increase of salt concentration, the concentration of reactive B species also increases in the solution, improving the polymer feature of SEI.

Solution ^1^H NMR spectra of SEIs from different electrolyte are shown in Figure 2d. In conventional carbonated‐based electrolyte, the monomeric SEI components of lithium ethylene monocarbonate (LEMC), ethylene glycol bis(methyl carbonate) (EGBMC) and residual solvent (methyl, MeOH and ethylene glycol, EG) can be easily observed, which offer low Li^+^ diffusion and weak mechanical strength.^[^ ^44^ ^]^ The SEIs formed in LiDFOB‐based ether electrolyte become more similar, while a small peak located in ≈3.47 ppm appears in 5 m DE electrolyte under NCM/Cu configuration, which cannot be found in Li/Cu configuration. We further perform the spectra of polyethylene oxide (PEO) and found out the same peak around ≈3.48 ppm, attributing to the H atoms in –(CH_2_–CH_2_–O)*_n_*– segment. As a result, the polyether character in SEI of 5 m DE NCM/Cu configuration is determined. This kind of polymeric SEI makes the interface more elastic to reducing the direct contact between deposited Li and electrolyte, thus improving the Li reversibility.

To confirm the solvation structure of 5 m DE, Raman spectra of electrolytes with different salt concentration in pure DME or DME/HFE mixtures are carried out. As shown in Figure 2e, the vibration bonds of O–CH_3_ in free DME appear at 850 and 820 cm^−1^. With the amount of LiDFOB increasing, the DME molecules prefer to coordinate with Li^+^ to form a LiDFOB‐DME pairs, reflecting as the diminishing peak of free DME and appearance of Li^+^‐coordinated DME (≈875 cm^−1^). After adding co‐solvent of HFE, the characteristic peak of Li^+^‐coordinated DME can still be observed without free DME generation, indicating that the solvation structure has been well preserved (Figure 2f).

**Figure 3:**
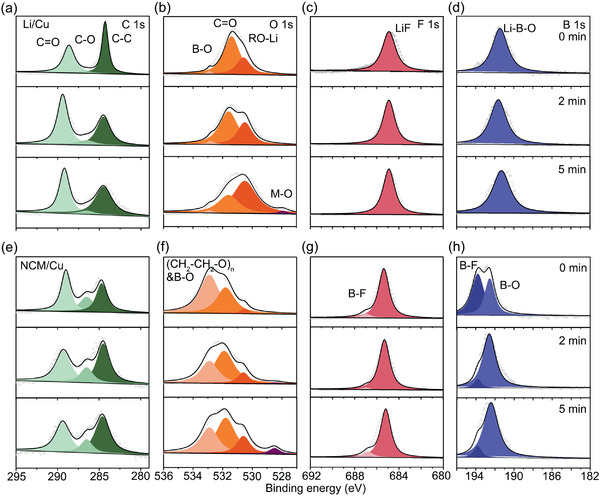
Characterizations of cycled Li anode using 5 m LiDFOB/DME/HFE system. a,e) C 1s; b,f) O 1s; c,g) F 1s; d,h) B 1s XPS spectra of the Li anode in Li/Cu and NCM/Cu configuration after 30 cycles.

The large differences of SEI in Li/Cu and NCM/Cu configuration are further studied using X‐ray photoelectron microscopy (XPS) and cryoelectron microscopy (Cryo‐EM). XPS depth profiling analysis on Li metal anode was performed to understand the detailed SEI formation under different electrochemical window. As shown in **Figure** **3**a,e, the in‐depth C 1s XPS spectra for both systems present three peaks including carbonyl group (C=O, 289.0 eV), polyether carbon (C–O, 286.5 eV) and hydrocarbon (C–H/C–C, 284.5 eV), respectively. However, the SEI cycled in NCM/Cu cell contains higher content of (–CH_2_–CH_2_–O–)*_n_* species, reflecting as a more obvious peak of polyether carbon in surface and subsequent etching, which indicates that the polymeric species exists in the entire SEI. The polymeric feature of NCMCu‐SEI is also demonstrated in O 1s XPS spectra. The SEI cycled in Li/Cu cells consists of C=O (531.7 eV) and RO–Li (530.5 eV) species with neglectable B–O signal, indicating that the organic phase in LiCu‐SEI is mostly derived from the decomposition of DME. As a comparison, the lower peak of RO–Li and new peak of B–O and (–CH_2_–CH_2_–O–)*_n_* can be clearly observed in NCMCu‐SEI, which can be attributed to the reactions between electron‐deficient B and electron‐rich species like RO–Li (Figure 3b,f). Moreover, after etching for 5 min, a small peak of M–O arises in NCMCu‐SEI (528.5 eV), showing the existence of metal oxides in deeper SEI. The B 1s and F 1s spectra reveal the different decomposition product of LiDFOB in different operation voltage. In LiCu‐SEI, only the high peak of LiF (684.9 eV) and Li–B–O (≈191.8 eV) can be observed in all depth while the additional peaks of F–B (687.0 eV) and B–F (≈194.0 eV) can both be detected in NCMCu‐SEI (Figure 3c,d,g,h). These results are also in accordance with the mechanism we proposed above.

## 4 Electrode Interface Analysis and Li Electrodeposition Morphology

**Figure 4:**
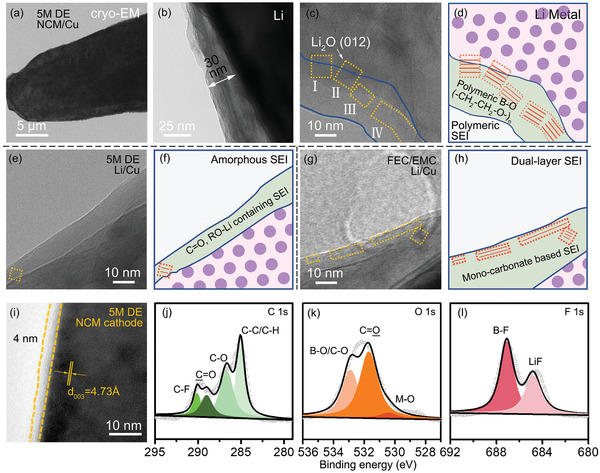
Observation of cathode electrolyte interface (CEI) and solid‐electrolyte interface (SEI). a–d) Nanostructure of deposited Li with high‐efficacy SEI in 5 m LiDFOB/DME/HFE, NCM/Cu configuration imaged by cryo‐EM. e,f) Amorphous monomer organic SEI layer in 5 m LiDFOB/DME/HFE, Li/Cu configuration. g,h) Conventional dual‐layer SEI layer in FEC/EMC system in Li/Cu configuration. i) HRTEM images and j–l) XPS spectra of NCM811 cycled in 5 m LiDFOB/DME/HFE electrolyte.

High‐resolution cryoelectron microscopy (cryo‐EM) was conducted to reveal the veil of SEI layer formed on the Li metal anode. The naturally formed SEI is made up of an amorphous organic phase with several crystalline randomly buried inside, which is generally nonhomogeneous with respect to both thickness and composition named mosaic‐like SEI. The composition and structure of SEI produced by different electrolytes are different, which will further induce the degradations of various performance of the Li metal anode. As shown in **Figure** **4**a, the deposited lithium of 5 m DE NCM/Cu cell shows a large granular size more than 10 µm with less tortuosity, which provides a good structural electronic connection while deposition compared with the one of FEC/EMC (Figure S9, Supporting Information). The thickness of as‐formed SEI layer is about 30 nm. The outer layer with amorphous structure comprises high molecular weight polyether and tri‐coordinated B species while inner layer shows a Li_2_O 〈111〉 crystalline structure (Figure 4b,c). The scheme and detailed information of SEI are shown in Figure 4d and Figure S10 (Supporting Information), which is consistent with the observation of XPS and ATR‐FTIR. The increasing concentration of LiDFOB salt will force the oxidation byproduct of DFOB^−^ anion into the inner‐Helmholtz layer to compete with solvent molecules, therefore the proportion of inorganic substances in inner SEI increase. This kind of multilayer SEI structure offers two merits for uniform Li ions transportation. 1) The outer organic polyether layer can provide rapid Li‐ion diffusion through local motion of polymer segments (like PEO) as well as flexibility to endure the large expansion. 2) The inner Li_2_O inorganic crystalline can directly prevent the electron tunneling from anode to electrolyte, further suppressing the electrochemical reduction of electrolyte components. In Li/Cu configuration, due to the low operation voltage, DFOB anion partially reduces with little polymeric ability, reflecting as an amorphous C=O, RO‐Li based organic SEI with little Li_2_O crystal (Figure 4e,f). This is the reason why 5 m DE shows different Li reversibility in Li/Cu and NCM/Cu configurations. This kind of NCMCu‐SEI also has large difference from the conventional solvent‐based SEI under low lithium salts concentration (e.g., 1 m LiPF_6_ in FEC/EMC), as shown in Figure 4g,h. The SEI formed in FEC/EMC system also shows a dual‐layer structure with outer Li_2_O and inner amorphous layer (mainly LEMC and other monocarbonate), which manifests as an opposite structure compared with that formed in 5 m DE electrolyte, indicating that the organic phase of SEI with polyether property is the main factor for reversible Li electrodeposition.

High‐resolution transmission electron microscopy (HRTEM) and XPS were carried out to probe the CEI structure formed on the NCM811 cathode in different electrolyte. Compared with pristine cathode with ordered rhombohedral phase, the cathode cycled in FEC/EMC electrolyte shows a rough and fuzzy surface with thin CEI covered (Figure S11, Supporting Information). Two kinds of lattice spacing of 4.73 and 2.37 Å are observed, which correspond to the 〈003〉 plane of initial layered structure and 〈111〉 plane of NiO rock‐salt phase, reflecting an irreversible structural degradation from Ni^4+^ to Ni^2+^ under high voltage charging. As a comparison, a conformal and amorphous CEI with a thickness of ≈4 nm is observed after cycling in 5 m DE electrolyte, which facilitates cathode protection (Figure 4i). XPS spectra show that the CEI formed in 5 m DE contains higher B–F and C=O species due to the decomposition of LiDFOB (Figure 4j–l).

**Figure 5:**
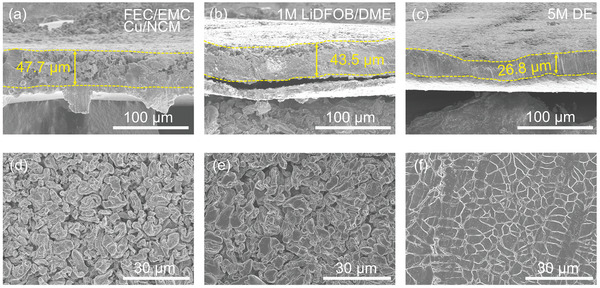
Deposition morphologies of 5 mAh cm^−2^ Li using different electrolytes in NCM/Cu configuration. Cross‐sectional and top‐view images of deposited Li in a,d) FEC/EMC system. b,e) 1 m LiDFOB/DME system. c,f) 5 m LiDFOB/DME/HFE system.

The morphology of deposited Li metal in different electrolytes was identified utilizing scanning electron microscopy (SEM) technique. The NCM/Cu cells with different electrolytes were first charged to 4.4 V to obtain a 5 mA h cm^−2^ deposited Li in first cycle. The cross‐sectional images of deposited Li in FEC/EMC and EC/DEC electrolyte are shown in **Figure** **5**a and Figure S12 (Supporting Information), that the thicknesses are almost twice compared to theoretical value (25 and 47.7 µm for FEC/EMC, and 68.4 µm for EC/DEC). Top‐view SEM images show the porous and dendritic morphology with high tortuosity in both electrolyte system, indicating loose structure with large void in deposited Li (Figure 5d). It also means that the small molecular weight species of C–H and aliphatic C–O–C cannot effectively protect the Li electrodeposition due to its low mechanical strength and uneven ionic flux. Although the lithium electrodeposition becomes larger in Li/Cu cell using 5 m DE, the increasing thickness of 45.1 µm can still be observed due to the poor SEI composition. The conditions become different in NCM/Cu cell with the increasing concentration of LiDFOB. The thickness of deposited Li decreases to 43.5 and 35.0 µm in 1 and 3 m LiDFOB/DME system. When 5 m DE is used, the thickness of electrode further decreases to 26.8 µm (Figure 5b,c), which is very close to the theoretical value of 25 µm, reflecting that the polymeric SEI layer induces tight deposition of lithium grain. The top‐view SEM image demonstrates the view that lithium particles grow compactly together to form a flat and seamless interface (Figure 5e,f; Figure S13, Supporting Information). As shown in Figure S14 (Supporting Information), after long‐time cycling in NCM/Cu, lithium metal is completely stripped away, and the SEI collapses without obvious crack. It is this excellent integrity that enables the lithium plate into SEI again in next cycle, thereby reducing the generation of new SEI and improving the reversibility of lithium.

## 5 Battery Cycling Performance under Harsh Condition

To prove the superiority of high‐efficacy SEI in practical condition, the requirements like high cycling current density (>0.3 C), high‐voltage (>4.3 V), high‐areal‐capacity (>4 mAh cm^−2^), lean electrolyte (*E*/*C* < 3 g Ah^−1^), and ultrathin Li foils (*N*/*P* < 2) should be met to increase energy density to 300 Wh kg^−1^. Low *N*/*P* (negative to positive electrode capacity ratio) and *E*/*C* (electrolyte weight to cathode capacity ratio) are two significant indicators for high‐energy density. However, few strategies have met these requirements due to the irreversible Li loss in harsh condition. Especially under high cathode loading with high current density, the deposition morphology is closely related to the quality of SEI. In full cell test, we utilize limited Li source of 9.02 mAh cm^−2^ (Figure S15, Supporting Information) to pair with aggressive cathode NCM811 (active mass loading: 21.5 mg, ≈5 mAh cm^−2^) under lean electrolyte and ≥4.3 V condition to evaluate the Li utilization and high‐voltage durability. As‐fabricated cells show a high energy density of ≈350 Wh kg^−1^ based on the mass of all cell components (Table S2, Supporting Information), which is promising to be utilized in electrical vehicles.

**Figure 6:**
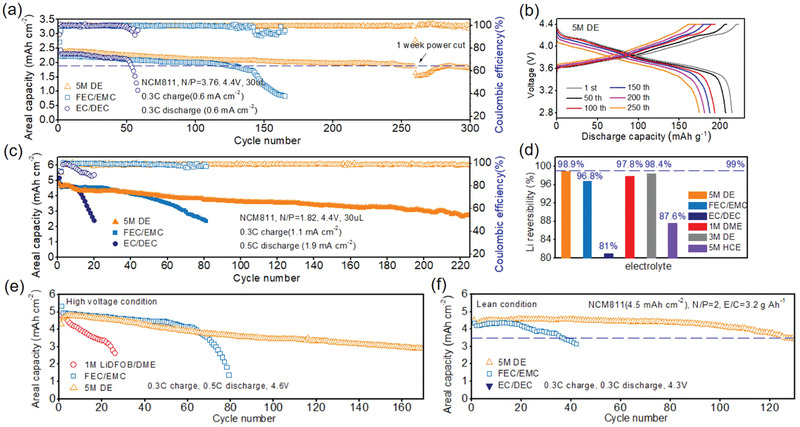
Electrochemical behavior of high‐voltage Li/NCM811 cells under harsh condition. a) Cycling performance of Li/NCM811 cells in three electrolytes with an *N*/*P* ≈ 3.76 (9 mAh cm^−2^ Li, 30 µL electrolyte) and b) corresponding voltage profiles of 5 m DE electrolyte sample. c) Cycling performance of Li/NCM811 cells in three electrolytes with an *N*/*P* ≈ 1.82 under 0.3C charge, 0.5C discharge condition. d) Calculated Li reversibility through full cell cycling performance in different electrolytes. e) 4.6 V cutoff voltage test for ether‐based and carbonate‐based electrolyte (9 mAh cm^−2^ Li, 30 µL electrolyte). f) ≈350 Wh kg^−1^ coin cell prototype using low *E*/*C* ratio of 3.2 g Ah^−1^.

First, the cells with a medium cathode areal loading of ≈2.4 mAh cm^−2^, thin Li anode, and excess electrolyte were assembled (*N*/*P* ≈ 3.76, **Figure** **6**a). The cells using conventional EC/DEC and FEC/EMC show short stable lifetime of 50 and 140 cycles, after which a sudden capacity drop can be observed due to the exhaustion of active Li. In contrast, the cell cycled in 5 m DE offers an 80% capacity retention after 303 cycles along with small cell overpotential increase (Figure 6b). The condition becomes more serious when *N*/*P* ratio decreases to 1.82. As shown in Figure 6c, the cell using EC/DEC electrolyte can only survive ten cycles at 0.3C charge/0.5C discharge rate before a sudden failure occurs. Adding FEC into carbonated electrolyte can improve the electrode/electrolyte stability and Li reversibility, reflecting as a prolonged lifespan of 80 cycles. However, the decreasing CE (<97% after 65th cycle) suggests the depletion of active Li in electrode, which was further demonstrated by cross‐sectional SEM (Figure S16a, Supporting Information). Impressively, the cell using 5 m DE exhibits a ultralong lifetime for more than 220 cycles without sudden drop (Figure S17a, Supporting Information), indicating a high Li utilization as well as less side reactions. To probe the electrode evolution after cycling, the cells were disassembled and washed with DME solution to remove the impurity. The Li anode cycling in FEC/EMC system shows a large volume expansion from 45 to 187.1 µm while the one cycling in 5 m DE just increases to 67.6 µm (Figure S16b, Supporting Information). The fine morphology stability may be attributed to the compact Li deposition behavior brought by B‐O based polymeric SEI layer. In addition, the cells using different concentration LiDFOB were also performed, as shown in Figure S17b (Supporting Information). The cycling performance is improved with the increase of salt concentration, which can be reflected by an enhanced and stable CE in full cell. It is worth noting that directly using high‐concentration LiDFOB electrolyte (5 m HCE) without diluting will bring even worse result than conventional FEC/EMC electrolyte, which may be related to the low Li^+^ transport kinetic brought by poor separator wettability and severe side reaction between excess LiDFOB and lithium metal. The estimate Li reversibility of different electrolytes is listed in Figure 6d, from which the Li anode cycled in 5 m DE electrolyte with polymeric SEI offers the highest CE of 98.9% under such a high current density and areal capacity.

The 4.6 V cutoff charge voltage was performed to evaluate the stability of LiDFOB‐based ether electrolyte (Figure 6e; Figure S18, Supporting Information). High cutoff voltage often gives rise to oxidative decomposition of conventional electrolytes, especially for the electron‐rich ethers. In electrolyte with low salt concentration of 1 m LiDFOB, large quantity of free DME molecules will decompose on cathode when the voltage exceeds 4.2 V, reflecting as a low CE in full cell (≈98%) and fast capacity degradation within 30 cycles. Fluorinated carbonated electrolyte possesses high voltage endurance due to the presence of electron withdrawing F groups, exhibiting a highest CE of 99.55% within 60 cycles. As a comparison, 5 m DE shows a lifetime of more than 160 cycles with an average CE of 99.23%, indicating that the coordinated DME molecules are stable under high voltage condition.

More impressively, when the amount of electrolyte decreases to industrial standard of 3.2 g Ah^−1^, the lifetime of cell using FEC/EMC electrolyte largely decreases to 38 cycles with 80% capacity retention. Such fast decay can be ascribed to electrolyte exhaustion caused by severe SEI formation between reactive electrolyte and fresh lithium metal. In contrast, the cell using 5 m DE possesses a long lifespan more than 129 cycles with a high energy density of ≈350 Wh kg^−1^ under 0.3C charge/discharge condition (Figure 6f; Figure S19, Supporting Information). This result is one of the best battery performances up to date under practical conditions when compared with other literature (Table S3, Supporting Information), indicating an outstanding sustainability of such high‐efficacy SEI for longtime operation.

## 6 Conclusion

In this work, an electrolyte‐derived polyether/coordinated borates containing SEI has been conducted by using 5 m LiDFOB/DME/HFE electrolyte at full cell condition. We found that the CE in Li/Cu cell is not completely equal to the utilization rate of lithium in the actual full battery cycling, which is also related to oxidation potential of electrolyte components. The detailed oxidation mechanism of concentrated LiDFOB on lithium metal anode has been investigated by DFT calculation and experiment. Under high voltage conditions, LiDFOB salts are oxidized to form electron‐deficient B species and further coordinate with other anions to induce cross‐linking reactions. Cryoelectron microscopy, NMR, XPS, and ATR‐FTIR observations reveals that the outer SEI layer is an amorphous polyether/tri‐coordinated borates polymeric organic phase, while the inner layer is a robust inorganic crystalline with Li_2_O lattice. It is this sustainable and robust SEI that offers a high Li reversibility in both NCM/Cu cells and full cells. In the harsh conditions of high cutoff voltage of 4.4 V and low *N*/*P* (2.0) and *E*/*C* (3.2 g Ah^−1^) ratios, the cells using diluted electrolyte show the impressive performance with 98.9% Li reversibility. Our vital findings provide an innovative way to manipulate the organic phase in SEI for high‐performance lithium metal‐based batteries.

## 7 Experimental Section

##### Simulation Details

The reaction mechanism was studied through density functional theory (DFT) calculations using the Gaussian 09W software (http://gaussian.com/). The Gibbs free energy *G* of each compound in reactions is a sum of the thermal correction to Gibbs free energy *Gcorr* and electronic energy *E*. *Gcorr* was obtained by the structure optimization and frequency analysis using the B3LYP functional method and the 6–31g(d) basis set, as well as the frequency correction factor 0.977, temperature 303.15 K and SMD solvation model for DME environment with the static dielectric constant eps  =  7.2 and dynamic dielectric constant epsinf  =  1.9033. *E* was obtained through the calculations of single point energy using the same DME solvation environment, M062X functional method and 6–311g(d,p) basis set.

##### Materials

Ultrathin Li metal foil of 9.02 mA h cm^−2^ was purchased from China Energy Lithium Co., Ltd. LiNi_0.8_Co_0.1_Mn_0.1_O_2_ cathode (NCM811: conductive carbon: polyvinylidene fluoride binder, PVDF = 97:2:1, by weigh) was supplied by SAIC Motor Passenger Vehicle Corporation with a cathode loading about ≈4 and 5 mAh cm^−2^. Lithium difluoro(oxalato)borate (LiDFOB), lithium hexafluorophosphate (LiPF_6_), fluoroethylene carbonate (FEC), ethyl methyl carbonate (EMC), 1,2‐dimethoxyethane (DME) were bought from DodoChem. 1,1,2,2‐Tetrafluoroethyl‐2,2,3,3‐tetrafluoropropylether (HFE) was bought from TCI (Shanghai) Development Co., Ltd. To prepare the diluted 5 m LiDFOB DE electrolyte, 715 mg LiDFOB was first put into 1 mL DME to form a transparent solution, after which 4 mL HFE was carefully added into the solution. To prepare the diluted 3 m LiDFOB DE electrolyte, 429 mg LiDFOB was first put into 1 mL DME to form a transparent solution, after which 2 mL HFE was carefully added into the solution.

##### Electrochemical Measurement

LAND electrochemical testing system and Solartron Analytical Electrochemical Workstation were used to study the electrochemical performance of lithium metal‐based cells. The NCM811/Li cells were cycled to the cutoff voltage of 4.3, 4.4 and 4.6 V (vs Li/Li^+^), respectively. All the cells are fabricated in the Argon‐fill glovebox with H_2_O and O_2_ < 0.1 ppm. For the cells cycled under the harsh condition of 350 Wh kg^−1^, 11 µL electrolyte was added into each cell. A rest step of 2 min was added after each charge process under lean electrolyte conditions. Li/Cu cells were prepared by using Cu foils as counter electrode and cycled at 0–2 V for five cycles to fully remove the contamination, after which a fixed amount of Li was plated on Cu electrode and stripped until the cutoff voltage increased to 1 V. NCM/Cu cells were prepared by using Cu foils and NCM811 (≈4 mA h cm^−2^) as electrode and cycled at 2.8–4.4 V.

##### Characterization

The surface composition was confirmed by X‐ray photoelectron spectroscopy (ESCALAB 250Xi, Thermo Fisher Scientific Inc., USA). The morphologies of lithium anode were analyzed by SEM SU‐8010 (Hitachi) at 5 kV. High‐Resolution Transmission Electron Microscope was performed on JEM‐2100 at 200 kV. The Cryoelectron microscopy was carried out on Talos F200C 200 kV. Attenuated total reflection Flourier transformed infrared spectroscopy (ATR‐FTIR) was carried out on Nicolet5700. Raman spectra were carried out on ThermoFisher DXR SmartRaman. 1H‐NMR spectra were performed on a 600 MHz DirectDrive2 spectrometer. Before test, the SEI residue from four disks was washed with DME solution and peeled off into glass bottle and dried for 12 h under 45 °C and 3 h in vacuum. Thereafter, DMSO‐d_6_ solution was added and heated under 40 °C for 1 h to dissolve SEI.

## Conflict of Interest

The authors declare no conflict of interest.

## Author Contributions

S.‐Y.L. and Y.‐Y.L. conceived the idea and designed the experiments. S.‐Y.L. and Q.‐L.L. performed the DFT calculation. All authors contributed in preparing the manuscript.

## Supporting information

Supporting Information
