# Supplementary material for: High‐Efficacy and Polymeric Solid‐Electrolyte Interphase for Closely Packed Li Electrodeposition
Source: Adv Sci (Weinh). 2021 Jan 29;8(6):2003240. doi: 10.1002/advs.202003240 (PMC7967057; doi:10.1002/advs.202003240)
Supplement: Supplementary file 1 — Supporting Information [file ADVS-8-2003240-s001.pdf]

© 2020 Wiley-VCH GmbH

ADVANCED SCIENCE
Open Access

Supporting Information

for *Adv. Sci.,* DOI: 10.1002/advs.202003240

High-Efficacy and Polymeric Solid-Electrolyte Interphase for Closely Packed Li Electrodeposition

Siyuan Li,[1] Qilei Liu,[2] Weidong Zhang,[1] Lei Fan,[1] Xinyang Wang,[1] Xiao Wang,[1] Zeyu Shen,[1] Xiaoxian Zang,[3] Yu Zhao,[3] Fuyuan Ma,[3] Yingying Lu[1,]*

High-Efficacy and Polymeric Solid-Electrolyte Interphase for Closely Packed Li Electrodeposition

Siyuan Li,[1] Qilei Liu,[2] Weidong Zhang,[1] Lei Fan,[1] Xinyang Wang,[1] Xiao Wang,[1] Zeyu Shen,[1] Xiaoxian Zang,[3] Yu Zhao,[3] Fuyuan Ma,[3] Yingying Lu[1,*]

[1]State Key Laboratory of Chemical Engineering, Institute of Pharmaceutical Engineering, College of Chemical and Biological Engineering, Zhejiang University, Hangzhou 310027, China

[2]Institute of Chemical Process Systems Engineering, School of Chemical Engineering, Dalian University of Technology, Dalian 116024, China

[3]Key Laboratory of Solar Energy Utilization & Energy Saving Technology of Zhejiang Province, Zhejiang Energy R&D Institute Co., Ltd., Hangzhou 311121, China

1. Supporting Figures

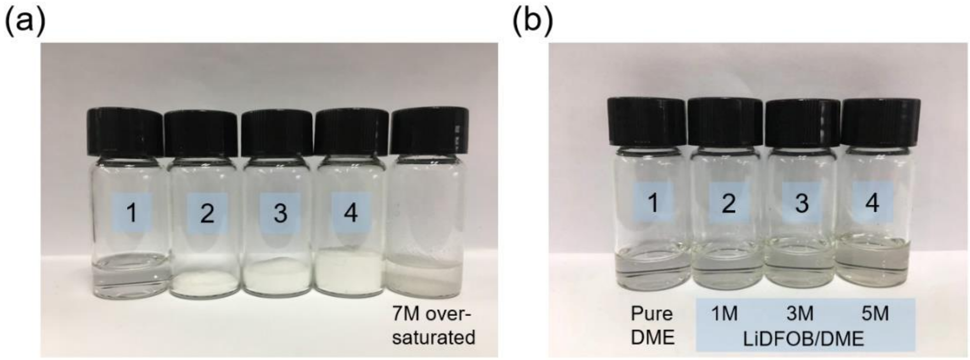

Figure S1. (a) Digital image of pure DME and different amount of LiDFOB salts. (b) Digital image of LiDFOB salt dissolving in DME electrolyte

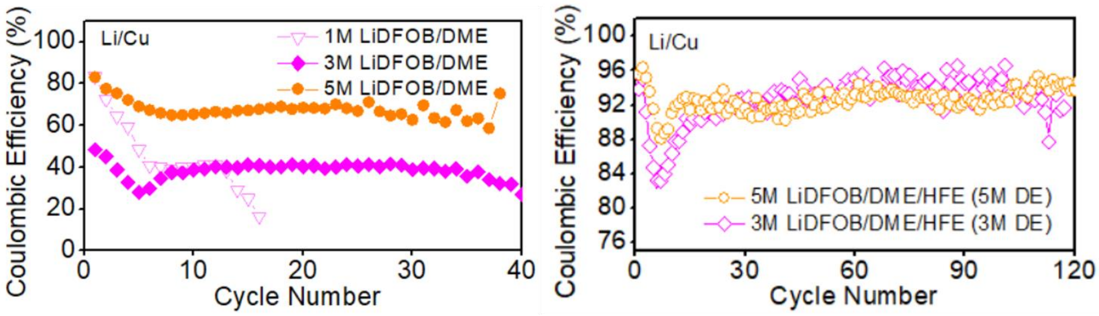

Figure S2. Li reversibility in Li/Cu configuration of three different concentration LiDFOB-based electrolytes.

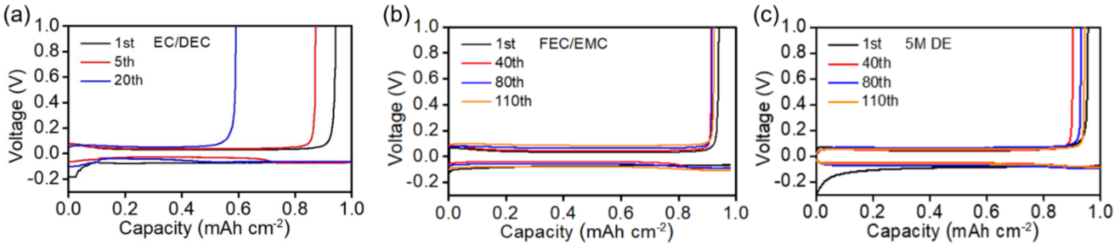

Figure S3. Voltage curves of Li/Cu cell using a) EC/DEC, b) FEC/EMC and c) 5M DE electrolyte.

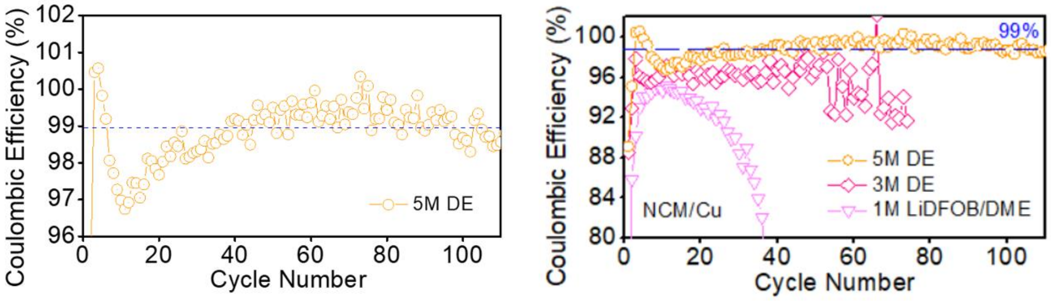

Figure S4. Real Li reversibility in NCM/Cu configuration of three different concentration LiDFOB-based electrolytes.

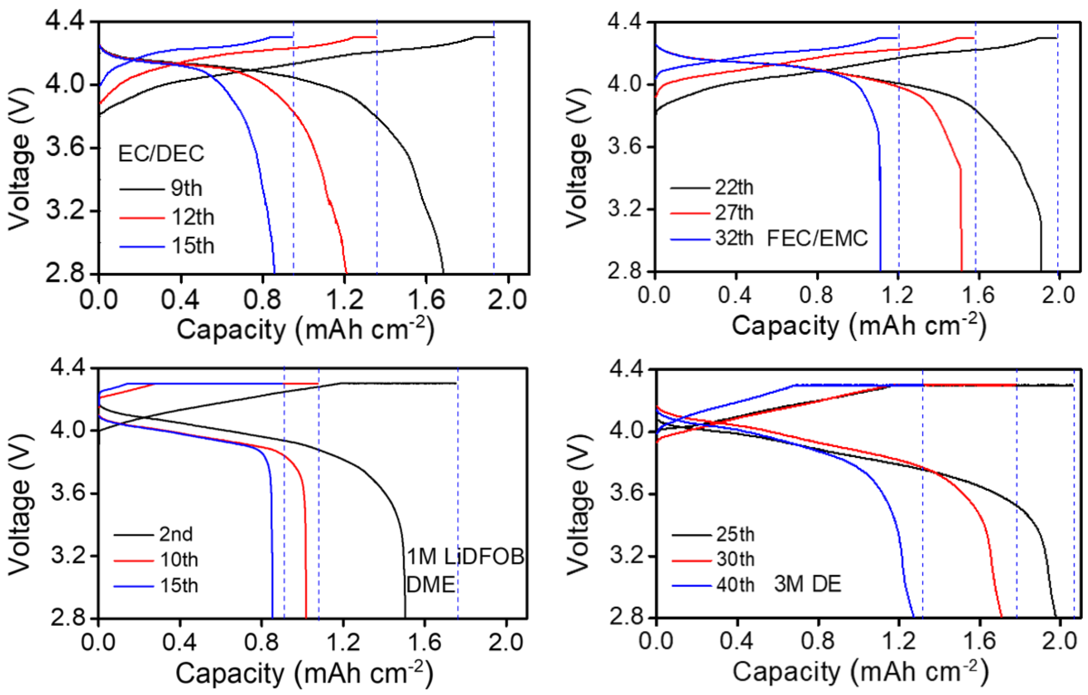

Figure S5. The detailed voltage curves of different electrolytes in NCM/Cu configuration.

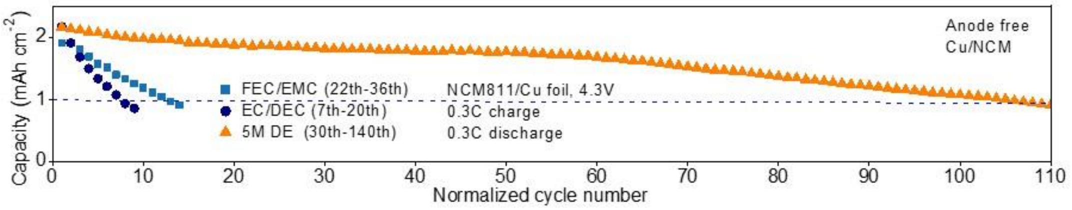

Figure S6. The normalized cycling performance of anode free Cu/NCM cell with a normalized initial capacity of ~2 mAh cm^{-2} (cathode loading ~4 mAh cm^{-2}).

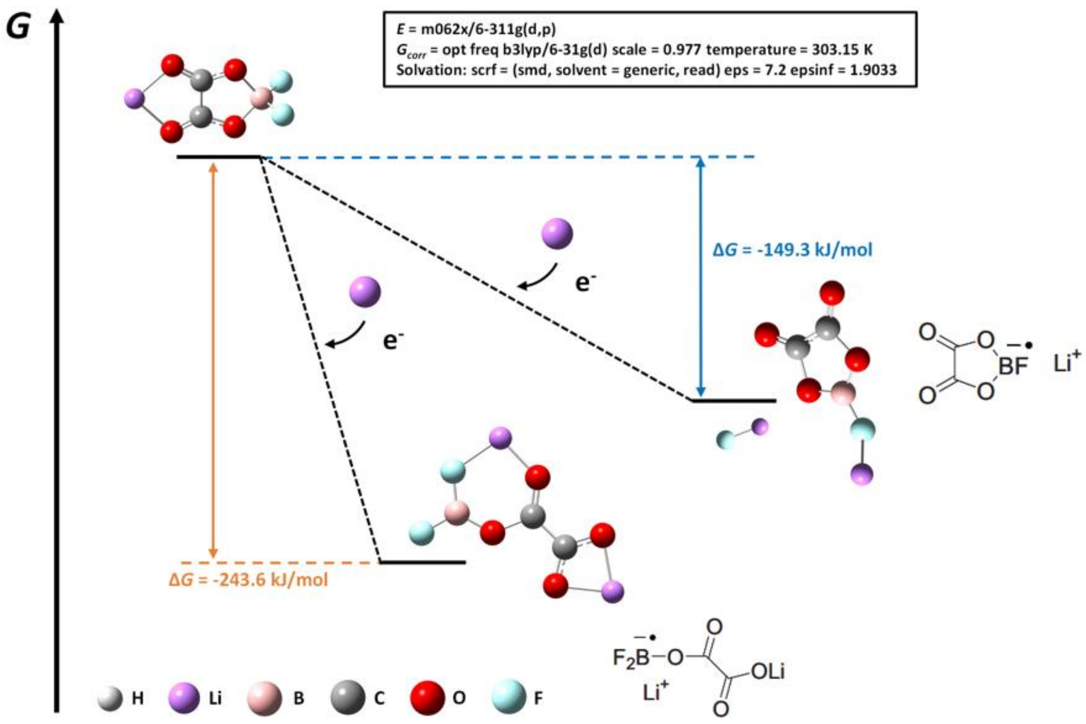

Figure S7. The proposed reducing pathways of LiDFOB on the Li metal anode in low voltage window.

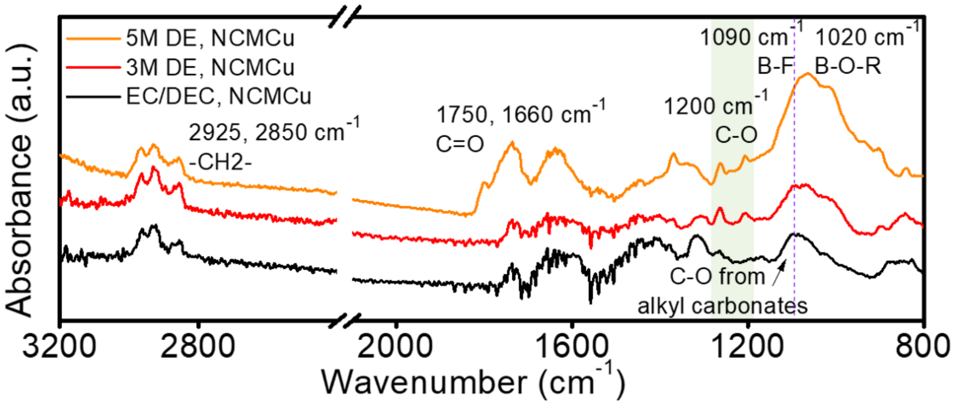

Figure S8. ATR-FTIR spectra of SEI residues induced by different electrolytes on Cu foils.

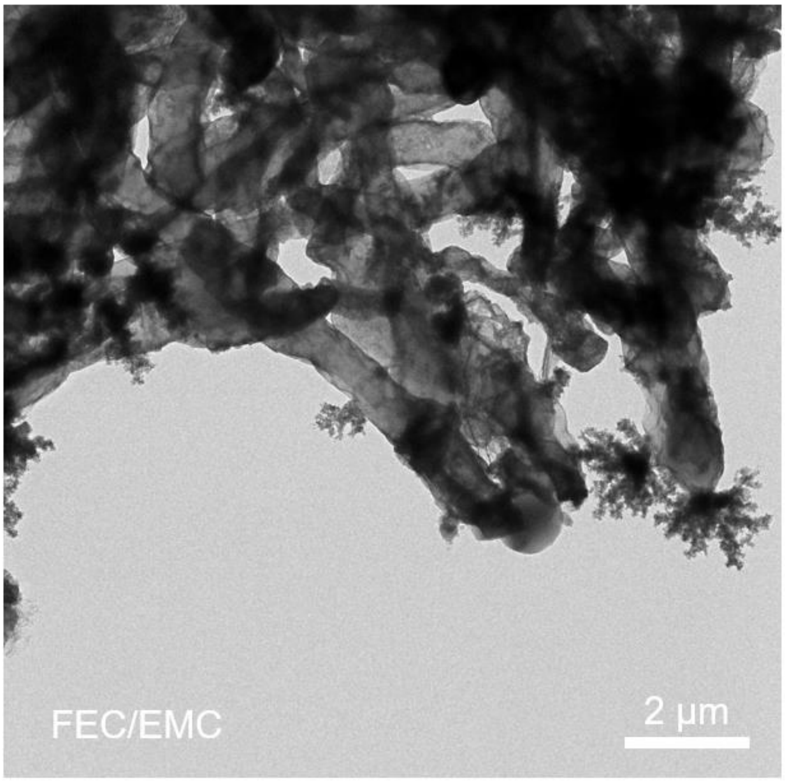

Figure S9. Cryo-EM images of deposited Li in FEC/EMC electrolyte.

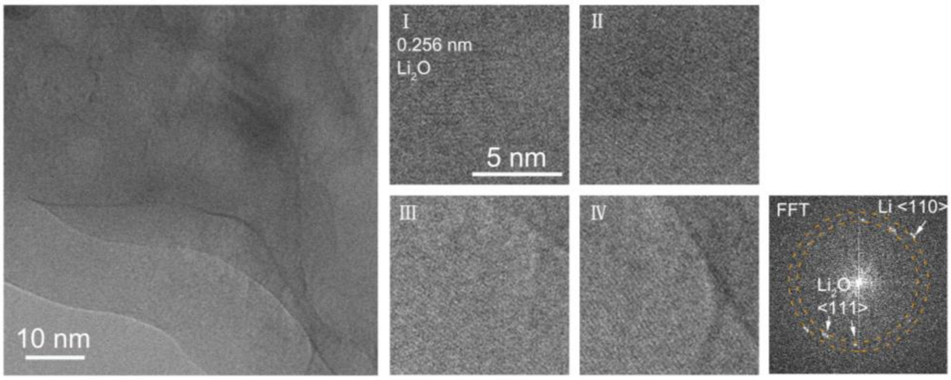

Figure S10. Enlarged cryo-EM images and FFT of SEI structure formed in 5M DE electrolyte in NCM/Cu cell. The Li_2O and Li crystalline is determined from FFT image (Gatan DigitalMicrograph 3.9). By using equation of R=2/L, the lattice spacing of Li_2O <111> and Li <110> can be obtained.

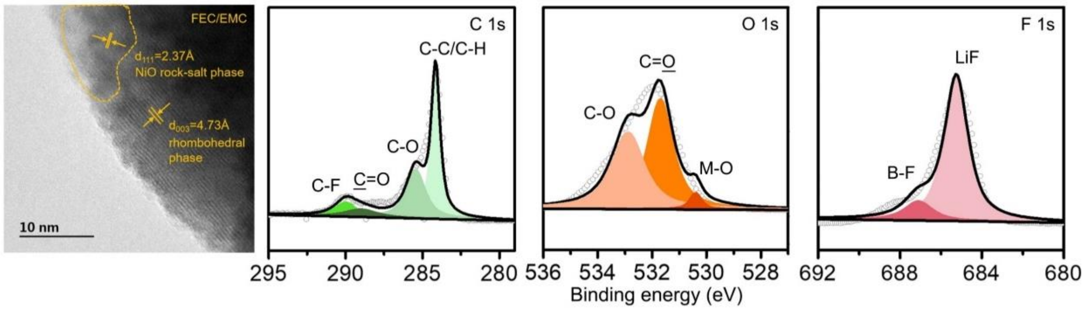

Figure S11. HRTEM images and XPS spectra of NCM811 cycled in FEC/EMC electrolyte.

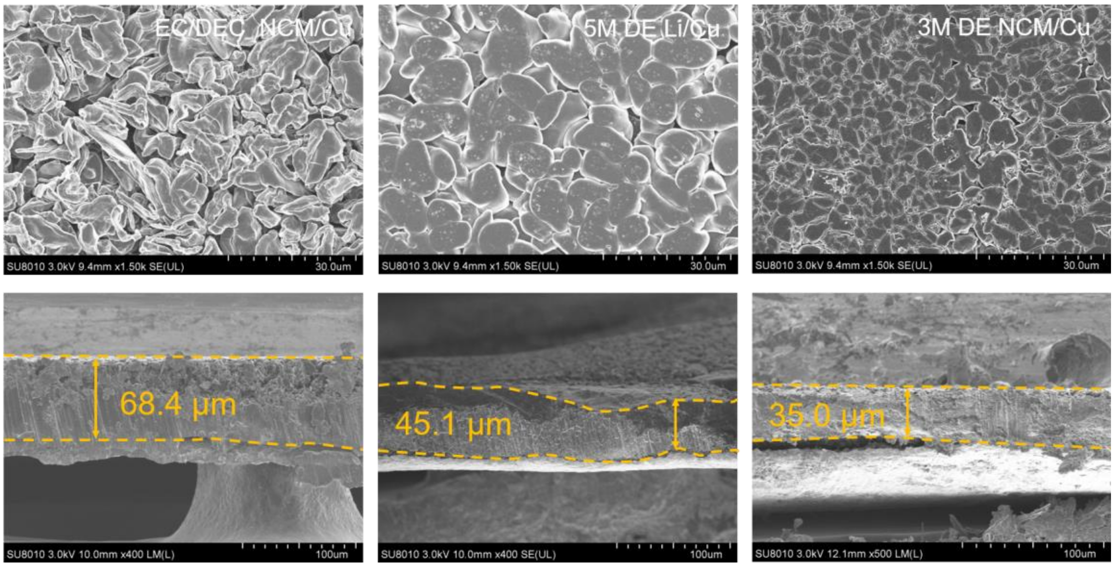

Figure S12. Cross-sectional and top-view deposition morphologies of 5 mAh cm^{-2} Li using different electrolytes in NCM/Cu or Li/Cu configuration.

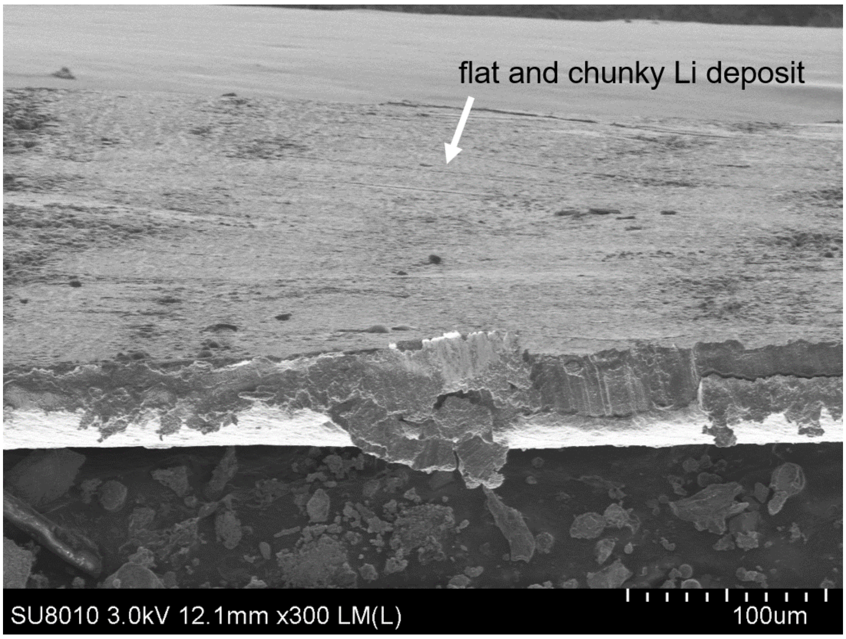

Figure S13. Cross-sectional deposition morphologies of 5 mAh cm^{-2} Li using 5M DE electrolyte in NCM/Cu configuration. As can be seen, flat and chunky Li deposit can be observed in all direction.

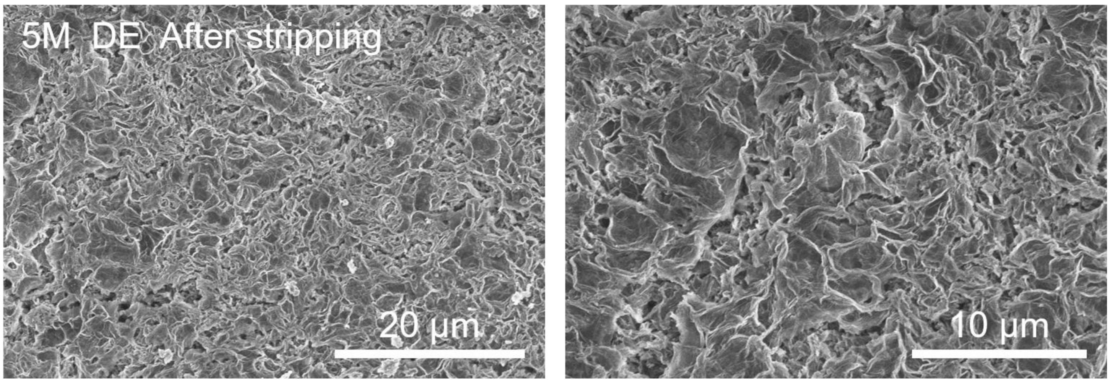

Figure S14. Top-view images of SEI residue after lithium metal is completely stripped away in 5M DE NCM/Cu cells after 86 cycles.

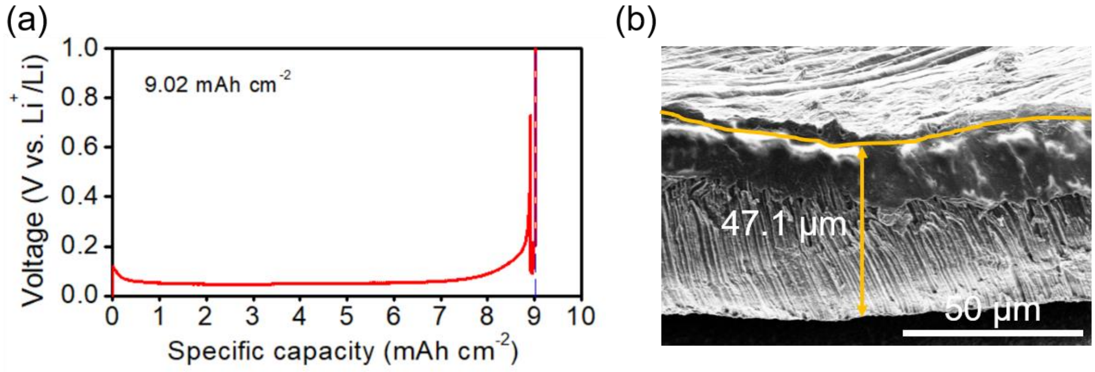

Figure S15. Characterization of ultrathin Li foil. a) Electrochemical Li stripping profile of ultrathin Li foil, showing a limited areal capacity of 9.02 mAh cm^{-2}. b) Cross-sectional SEM image of Li foil, indicating an active Li thickness of 47.1 μm.

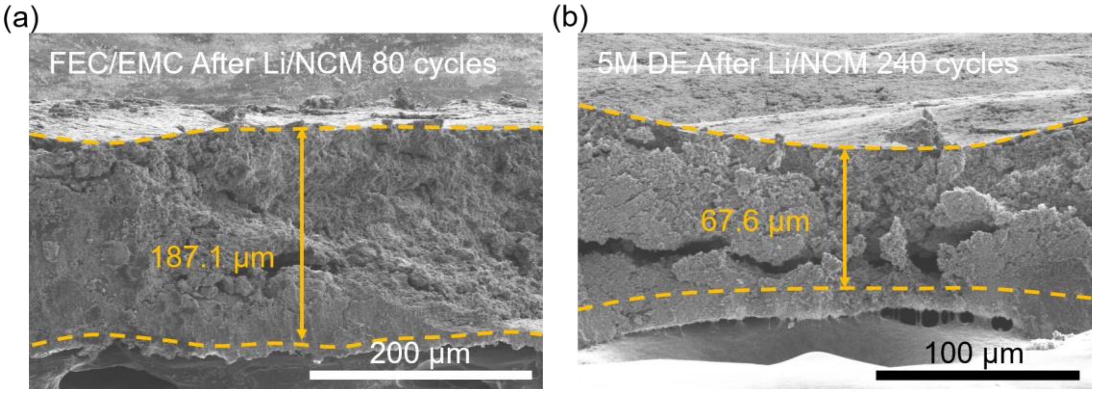

Figure S16. Cross-sectional images of thin Li foils after cycling in a) FEC/EMC and b) 5M DE electrolyte.

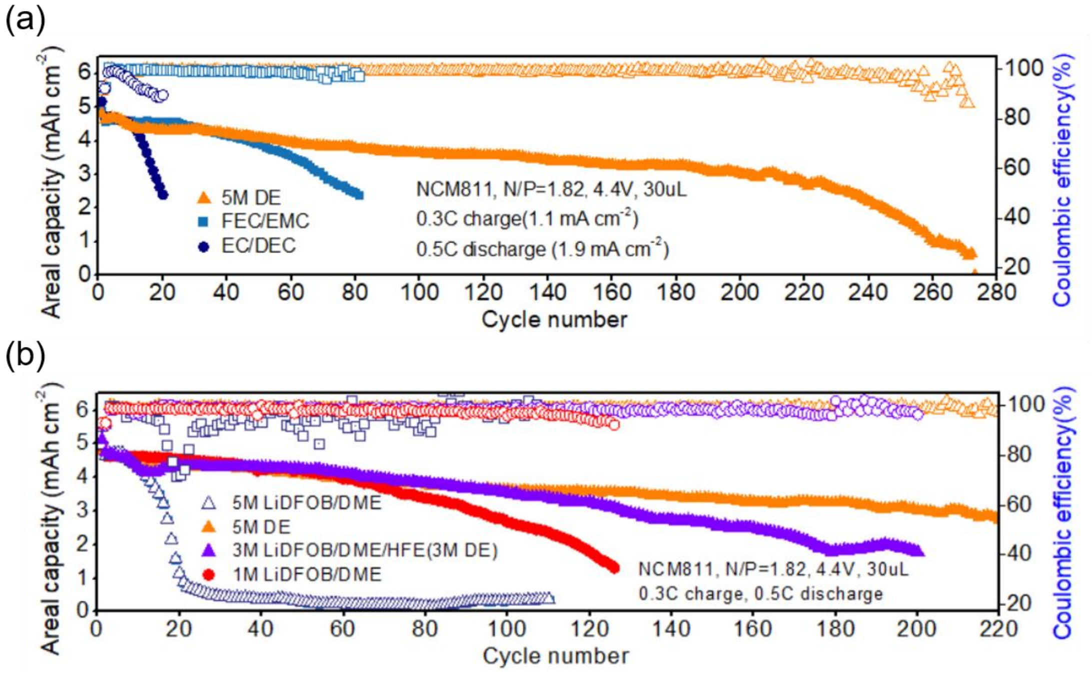

Figure S17. a) Full cycling performance of Li/NCM811 cells in three electrolytes with a N/P~1.82 under 0.3C charge, 0.5C discharge condition. b) cycling performance of Li/NCM811 cells in four LiDFOB-based electrolytes with a N/P~1.82 under 0.3C charge, 0.5C discharge condition.

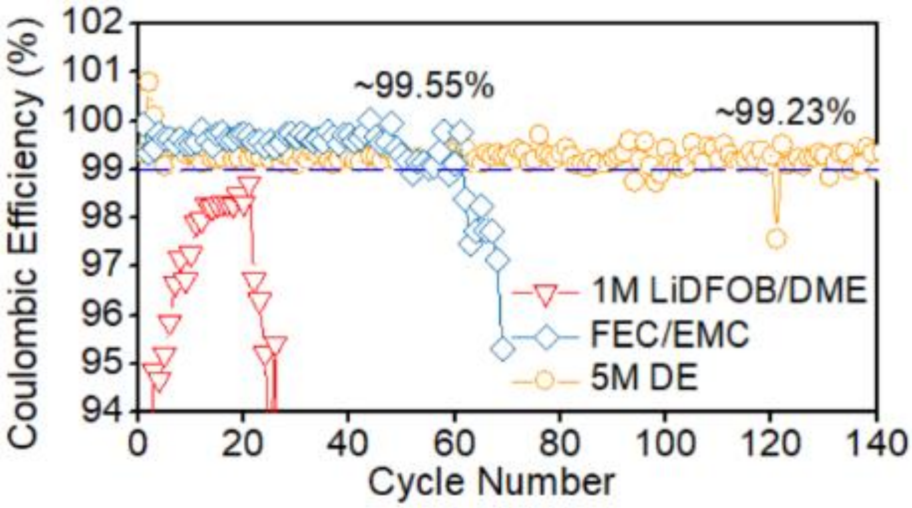

Figure S18. Enlarged image of CE in 4.6V Li/NCM811 full cells with different electrolyte.

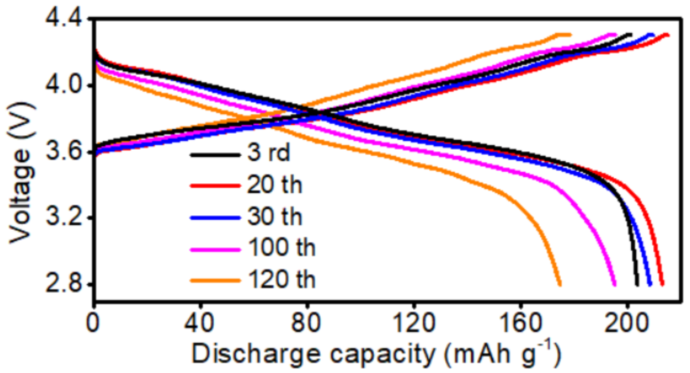

Figure S19. Voltage profiles of Li/NCM811 cell cycled in harsh lean condition in Figure 6f.

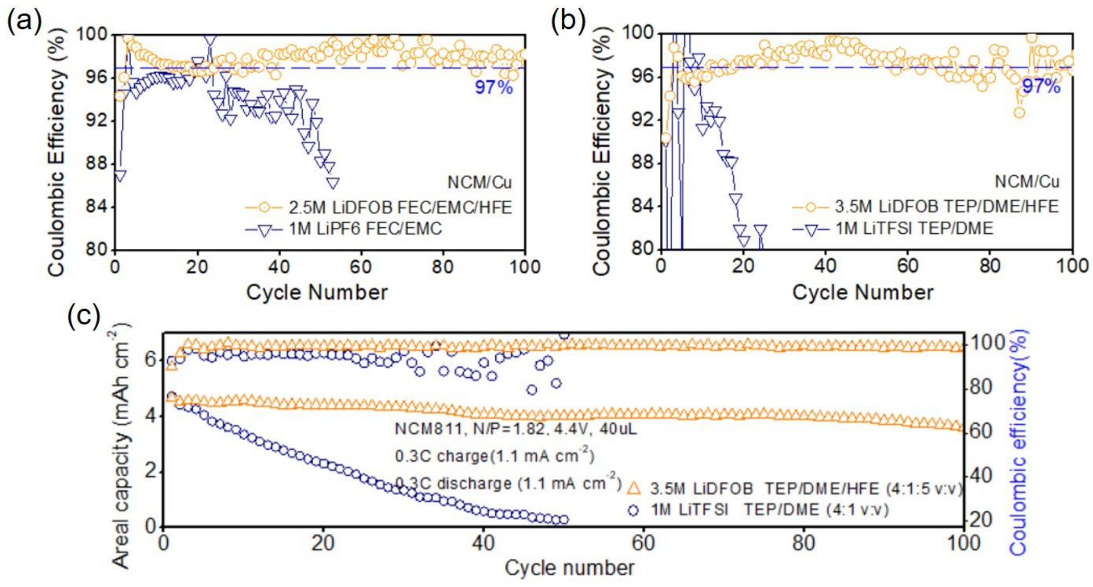

Supplementary Note 1. Performance of ester and phosphate-based electrolyte based on high concentration LiDFOB salt. a) Li reversibility in NCM/Cu configuration of 2.5M LiDFOB FEC/EMC/HFE (3:7:10 v:v, diluted to 1.25M) electrolyte and 1M $LiPF_6$ FEC/EMC (3:7 v:v), 0.3C charge, 0.3C discharge. b, c) Li reversibility in NCM/Cu configuration and full cell cycling of 3.5M LiDFOB TEP/DME/HFE (4:1:5 v:v, diluted to 1.75M) electrolyte and 1M LiTFSI TEP/DME (4:1 v:v), 0.3C charge,

0.3C discharge.

2. Supporting Tables

Table S1. Viscosity of different electrolytes and LiDFOB-based electrolytes

Type	Viscosity (mPa·s)
1M $LiPF_6$ in EC/DEC	4.164
1M $LiPF_6$ in FEC/EMC	2.909
1M LiDFOB in DME	2.244
5M LiDFOB in DME	26.74
5M LiDFOB in DME/HFE (5M DE)	3.799

Table S2. Parameters of 350 Wh kg^{-1} level coin cell prototype.

Parameter		Li/NCM in Fig 6f
Cathode areal capacity		4.5 mAh cm^{-2}
Anode areal capacity		9.02 mAh cm^{-2}
N/P ratio		2.0
E/C ratio		3.19 g Ah^{-1}
Discharge capacity		224.9 mAh g^{-1}
Average discharge voltage		3.78 V
Weight	Cathode (1 cm)	22.39 mg
	Al foil	3.3 mg
	Electrolyte	14.37 mg (11 μL)
	Separator	2.61 mg
	Anode	10.6 mg
	Total weigh	53.27 mg
Energy density		346.60 Wh kg^{-1}

Table S3. Summary of cycling performance of state-of-the-art Li/NCM811 system under harsh condition.

Ref.	Cathode loading (mAh cm^{-2})	N/P E/C	Cycle number	Charge/Discharge rate (C)	Energy density in coin cell (Wh kg^{-1})
This work	4.5 (NCM811)	2.0 / 3.19	129	0.3/0.3	346.6
LiFSI-1.2DME-3TTE[4]	4.2 (NCM811)	2.38 / 3	155	0.333/0.333	325 (without Cu)
Li-C Self-smoothing anode[5]	4.2 (NCM811)	1.5 / 3	200	0.2/0.3333	381
all-fluorinated electrolyte[6]	2 (NCM811)	1 / excess	120	0.5/0.5	N/A
fCNT-PRPAA network[7]	1.5 (LFP)	2.67 / excess	90	1 / 1	N/A
NCM622 pouch cell[8]	3.8	2.6 / 3	200	0.2 / 0.333	300
MTFP-FEC[9]	1.3 (NCM811)	2 / excess	80	0.5 / 0.5	N/A
LiDFOB/$LiBF_4$ dual salts[10]	16.1 mg cm^{-2}	Anode free	200	0.2 / 0.5	N/A

Reference

1. Ochterski J W. Thermochemistry in gaussian. Gaussian Inc, 2000: 1-19.
2. Alecu I M, Zheng J, Zhao Y, et al. *Journal of chemical theory and computation*, 2010, **6**, 2872-2887.
3. Marenich, A.V., Cramer, C.J., Truhlar, D.G. *Journal of Physical Chemistry B*, 2009, **113**, 4538-4543.
4. X. Ren, L. Zou, X. Cao, M. Engelhard, W. Liu, S. Burton, H. Lee, C. Niu, B. Matthews, Z. Zhu, C. Wang, B. Arey, J. Xiao, J. Liu, J. Zhang, W. Xu, *Joule*, 2019, **7**, 1662-1676.
5. C. Niu, H. Pan, W. Xu, J. Xiao, J. G. Zhang, L. Luo, C. Wang, D. Mei, J. Meng, X. Wang, Z. Liu, L. Mai and J. Liu, *Nature nanotechnology*, 2019, **14**, 594-601.

6. X. Fan, L. Chen, O. Borodin, X. Ji, J. Chen, S. Hou, T. Deng, J. Zheng, C. Yang, S. C. Liou, K. Amine, K. Xu and C. Wang, *Nature nanotechnology*, 2018, **13**, 715-722.
7. Yoo, D.- J., Elabd, A., Choi, S., Cho, Y., Kim, J., Lee, S. J., Choi, S. H., Kwon, T.- w., Char, K., Kim, K. J., Coskun, A. and Choi, J. W., *Advanced Materials,* 2019, **31**, 1901645.
8. C. Niu, H. Lee, S. Chen, Q. Li, J. Du, W. Xu, J. Zhang, M. Whittingham, J. Xiao and J. Liu. *Nature Energy*, 2019, **4**, 551-559.
9. J. Holoubek, M. Yu, S. Yu, M. Li, Z. Wu, D. Xia, P. Bhaladhare, M. Gonzalez, T. Pascal, P. Liu, and Z. Chen. *ACS Energy Letters*, 2020, **5**, 1438-1447
10. M. Genovese, A. J. Louli, R. Weber, C. Martin, T. Taskovic, and J. R. Dahn. *Journal of The Electrochemical Society*, 2019, **166**(14), A3342-A3347.
